# XLH Matters: an evolving programme to discuss new advances and share clinical experiences to improve patient outcomes

**DOI:** 10.1186/s13023-024-03387-4

**Published:** 2025-02-03

**Authors:** Lothar Seefried, Ferdinando Aliberti, Cathrine Alsaker Heier, Pedro Arango-Sancho, Martin Biosse Duplan, Sophia D. Sakka, Francesco Emma, Oliver Gardiner, Muhammad Kassim Javaid, Rui M. Ferreira-Santos, Adalbert Raimann, Kristen Rak, Judith S. Bubbear, Moira S. Cheung, Signe Sparre Beck-Nielsen, Gabriel T. Mindler, Agnès Linglart

**Affiliations:** 1 Orthopedic Institute, König-Ludwig Haus, University of Würzburg, Würzburg, Germany; 2 Craniofacial Unit, Neurosurgical Department, Santobono Children’s Hospital, Naples, Italy; 3 Pediatric Department, Oslo University Hospital, Oslo, Norway; 4 Department of Pediatric Nephrology, Hospital Sant Joan de Déu, Barcelona, Spain; 5 Department of Onco-Nephrology, Pediatric Cancer Center, Barcelona, Spain; 6 UFR Odontologie, Université Paris Cité, Paris, France; 7 Service de Médecine Bucco-Dentaire, Hopital Bretonneau APHP, Paris, France; 8 Division of Endocrinology, Metabolism, and Diabetes, First Department of Pediatrics, ENDO-ERN Center for Rare Pediatric Endocrine Disorders, National and Kapodistrian University of Athens Medical School, “Aghia Sophia” Children’s Hospital, Athens, Greece; 9 Mitera Children’s Hospital, Athens, Greece; 10 Division of Nephrology, Bambino Gesù Children’s Hospital—IRCCS, Rome, Italy; 11 International XLH Alliance, London, UK; 12 Nuffield Department of Orthopaedics, Rheumatology and Musculoskeletal Sciences, University of Oxford, Oxford, UK; 13 Evelina London Children’s Hospital/Guy’s and St Thomas’ NHS Foundation Trust, London, UK; 14 Division of Pediatric Pulmonology, Department of Pediatrics and Adolescent Medicine, Allergology and Endocrinology, Medical University of Vienna, Vienna, Austria; 15 Vienna Bone and Growth Center, Vienna, Austria; 16 Department of Oto-Rhino-Laryngology, Plastic, Aesthetic and Reconstructive Head and Neck Surgery and the Comprehensive Hearing Center, University of Wuerzburg, Würzburg, Germany; 17 Royal National Orthopaedic Hospital, Stanmore, Middlesex, UK; 18 Great Ormond Street Hospital, NHS Foundation Trust, London, UK; 19 Centre for Rare Diseases, Aarhus University Hospital and Department of Clinical Research, Aarhus University, Aarhus, Denmark; 20 Department of Pediatric Orthopaedics, Orthopaedic Hospital Speising, Vienna, Austria; 21 APHP, Endocrinology and Diabetology for Children, Bicêtre Paris Sud Hospital, Le Kremlin-Bicêtre, France; 22 APHP, Reference Center for Rare Disorders of Calcium and Phosphate Metabolism, Filière OSCAR, Paris, France; 23 APHP, Platform of Expertise for Rare Disorders Paris-Sud, Bicêtre Paris Sud Hospital, Le Kremlin-Bicêtre, France

## Expanding the XLH Matters programme

XLH Matters has become one of the ‘go-to’ annual meetings held in Europe for healthcare professionals (HCPs) from a wide range of specialties, across geographical regions, who treat people living with XLH. This programme brings HCPs together to hear about the latest research, discuss current clinical challenges and share experiences to inspire local practice and improve patient outcomes. A comprehensive summary of the key findings from XLH Matters 2022 is available in ‘XLH Matters 2022: Insights and recommendations to improve outcomes for people living with X-linked hypophosphataemia (XLH)’ [1]. The key objectives of the XLH Matters programme are listed in Box 1.

**Box 1:** Key objectives of the XLH Matters programme

Optimise the treatment and overall management of patients with XLH
Facilitate collaboration and knowledge sharing between a network of XLH experts
Share the latest clinical and real-world data in XLH
Discuss educational needs and identify key challenges that affect XLH management at international, regional and local levels

The third annual meeting welcomed 145 HCPs from 27 countries—the highest attended XLH Matters meeting so far. Clinicians with a range of specialties involved in the treatment of children and adults with XLH were present, including endocrinologists, nephrologists and rheumatologists, among others. The expert faculty specifically focused on three core topics based on suggestions from XLH Matters 2022, as well as new insights from the XLH field:

1. Improving health-related quality of life (HRQoL) for people living with XLH
2. Collaborating as a multidisciplinary team (MDT)
3. Continuing care for adolescents with XLH

A series of interactive sessions and workshops were held, with key outputs recorded on workmats (Table 1). Challenges and current knowledge gaps identified during faculty-led roundtable discussions were also recorded.

**Table 1:** XLH Matters 2023 workshop sessions

Workshop 1 Optimising outcomes: Improving HRQoL for people living with XLH	
*Part 1*	
Grouped attendees were asked to record their responses to the following three questions on a workmat: 1. What HRQoL outcomes do you currently measure? 2. What HRQoL outcomes do you think should be measured for people living with XLH? 3. What barriers do you encounter when measuring HRQoL?	
*Part 2*	
Attendees were split into paediatric and adult care providers, and asked to list the changes that they would implement in their clinical practice on the cards provided	

Workshop 2 Meet the XLH experts from the MDT: Dentist, radiologist, ear nose and throat specialist, and neurosurgeon	
From a list of three top tips provided by each of the XLH experts from the MDT, attendees individually voted for the top tip that they would consider applying to their clinical practice on workmats	

### Recent updates from the XLH field

**Fig. 1:**
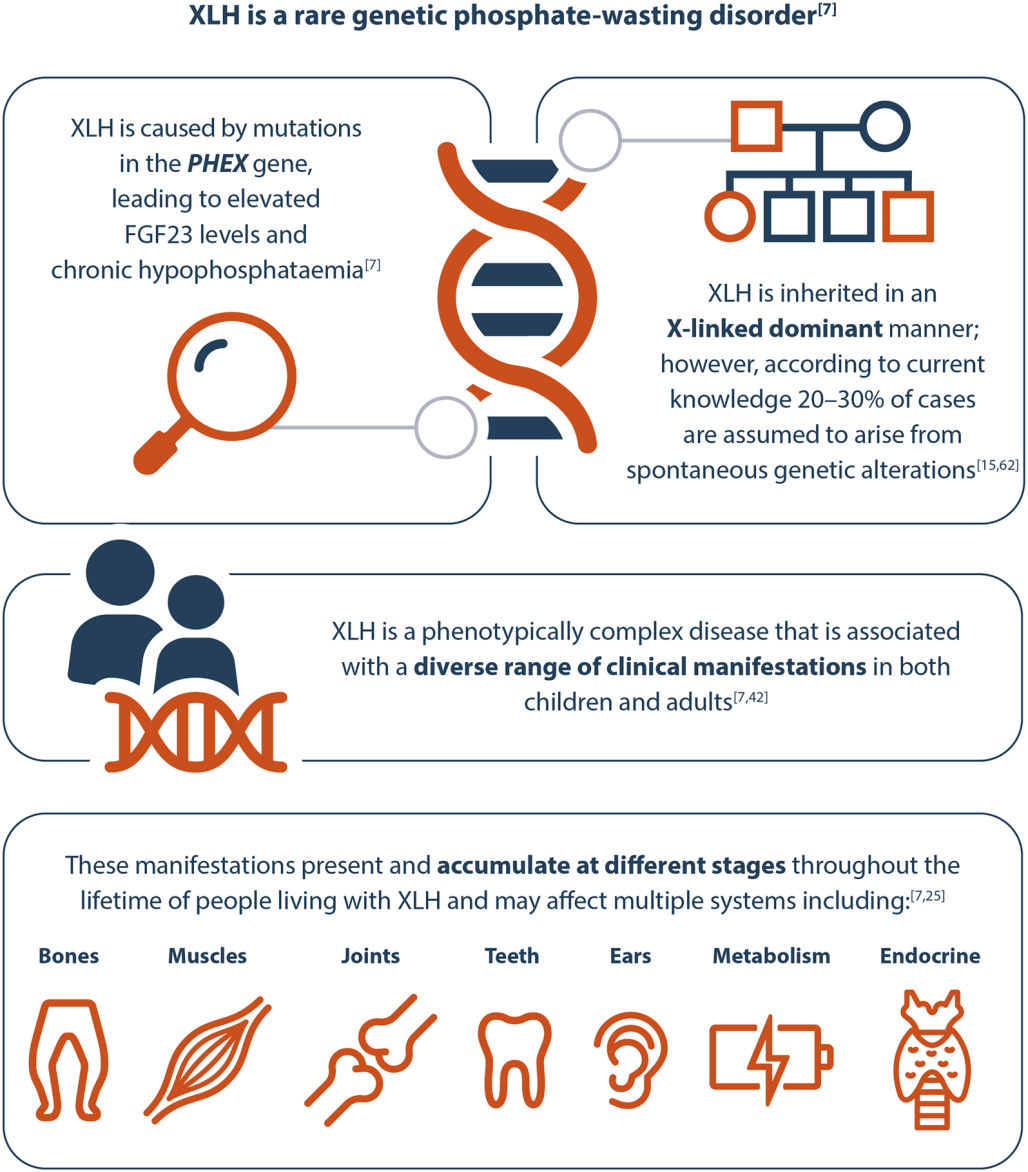
The pathophysiology and clinical manifestations of XLH. FGF23, fibroblast growth factor 23; PHEX, phosphate-regulating endopeptidase homologue on the X chromosomes; XLH, X-linked hypophosphataemia

A comprehensive session provided the latest updates from the XLH field. Ongoing scientific efforts to increase our understanding of XLH (Fig. 1) and to collate real-world data are vital for determining optimal treatment approaches for people living with XLH.

Since the approval of burosumab in the EU in 2020 for the treatment of XLH in children and adolescents (aged 1–17 years) with radiographic evidence of bone disease and adults, research has focused on the long-term impact of burosumab on XLH [2–4]. To explore the anticipated impact of burosumab on clinical sequelae in adults with XLH, eight global experts in XLH completed a questionnaire and participated in an online discussion platform to gain insight on the links between XLH pathophysiology and clinical implications [3]. The experts principally agreed that burosumab treatment is very likely to prevent the development of future fractures and reverse existing pseudofractures, and is expected to prevent the development of specific clinical and radiographic manifestations, such as enthesopathy, spinal stenosis, tinnitus and hearing loss in adults [3].

Clinical trial data evaluating the long-term use of burosumab in children and adults with XLH have recently been published [5, 6]. In an open-label trial, long-term burosumab treatment of 52 children (aged 5–12 years) with XLH for 160 weeks improved phosphate homeostasis (46% increase in serum phosphate from baseline; *p* < 0.0001) and rickets, as indicated by a significant decrease in total Rickets Severity Scores (RSS) from baseline (−0.9 ± 0.1; *p* < 0.0001) [5]. In a Phase III trial of 31 adults with XLH, improvements in patient-reported outcome measures (PROMs) and ambulatory function were reported in those who continued burosumab treatment from a 96-week study into a 48-week open label extension (OLE) [6]. Significant improvements were maintained in Western Ontario and McMaster Universities Osteoarthritis Index (WOMAC) stiffness and physical function measures, Brief Pain Inventory (BPI) and Brief Fatigue Inventory (BFI) scores and 6-min walk test (6MWT) distance (*p* < 0.05) [6]. Furthermore, an exploratory analysis highlighted the importance of continued treatment with burosumab, with clinical benefits lost in those who discontinued treatment between the initial study and the OLE [6].

Since XLH Matters 2022, there have also been many notable advances in our understanding of the disease mechanisms in XLH; highlighted in Box 2.

**Box 2:** Selected advances in the understanding of XLH pathophysiology

In a case-controlled study using ^31^P magnetic resonance spectroscopy, Kara et al. reported a significant reduction in the muscle performance and strength in patients with XLH compared with matched controls (*p* < 0.001). However, this was not associated with alterations in intramuscular phosphate metabolites at a resting state [8].
*Klotho* transcripts were significantly upregulated in the fourth ventricle of *Hyp* mice, the murine model of XLH, whereas *Atp1a1* transcripts were significantly downregulated in the third and lateral ventricle compared with controls (*p* < 0.05). These transcripts are involved in the production of cerebrospinal fluid in *Hyp* mice. Further research is needed to determine if these alterations contribute to cranial and spinal malformations in XLH [9].
A new *PHEX* gene locus-specific database (*PHEX* LSDB) has recently been made available and contains the largest collection of unique variants to date (870 unique variants as of April 2021). Analysis of the database indicated that *PHEX* does not appear to have a predominant variant associated with XLH, with many variants only appearing a few times in the database (Available at: https://www.rarediseasegenes.com) [10].

Recommendations to support clinicians in the diagnosis and management of XLH were first published by Haffner et al. in 2019, with updated guidance anticipated in 2025 [7]. In the interim period, it is imperative that clinicians share their expertise, research findings and best practice to optimise patient care. Accordingly, a group of ten European HCPs from seven different countries, experienced in treating people with XLH, convened to share their insights concerning the use of burosumab in children and adolescents [4]. Following virtual and collaborative working sessions, the expert group recommended prompt initiation of burosumab starting from infancy, according to the applicable licensing, especially in those with profound rickets (total RSS ≥ 2), and the continuation of burosumab treatment throughout adolescence and in adulthood, wherever possible [4].

To review the latest advances in XLH research, HCPs were asked to rate how well informed they felt (1 = not informed, 5 = very informed) prior to attending XLH Matters 2023. Survey results revealed that only 63% (77/122) of respondents considered themselves ‘informed (4)’ or ‘very informed (5)’. Conversely, 30% (37/122) considered themselves ‘neither informed or not informed (3)’ and 7% (8/122) considered themselves ‘poorly informed (2)’.

Therefore, continuous collaboration via network meetings, such as XLH Matters, can provide HCPs with a valuable platform to keep up to date, and share their knowledge and experience.

This supplement summarises the key outputs and discussions from the XLH Matters 2023 meeting, providing an interim resource to support clinicians by: (1) improving clinical practice to optimise patient outcomes; and (2) addressing gaps in published literature and guidelines.

## Living with XLH—Our patients' perspectives

The International XLH Alliance (IXLHA) is an association of patient groups for individuals affected by XLH, comprising more than 23 member organisations worldwide [11]. Their mission is to bring all the groups together to: amplify the patient voice of XLH and related disorders; set a global multidisciplinary standard of care and research; and ensure that all patients have access to the same management and treatment [11]. To align with the mission, IXLHA has developed 12 recommendations that can be used by patients to advocate for better care, which are complementary to Haffner et al.’s guidelines, ‘Clinical practice recommendations for the diagnosis and management of X-linked hypophosphataemia’ [7, 12].

At XLH Matters 2023, Oliver Gardiner, the Chair of the IXLHA, advised clinicians on how to identify gaps in XLH care, inform quality improvement and support the IXLHA’s campaign on best care. The aim of this section is to provide the patients’ perspective on the current standards of care for XLH and highlight how HCPs can support their patients to manage the disease burden more effectively.

### Patients’ insights on current XLH care

**Fig. 2:**
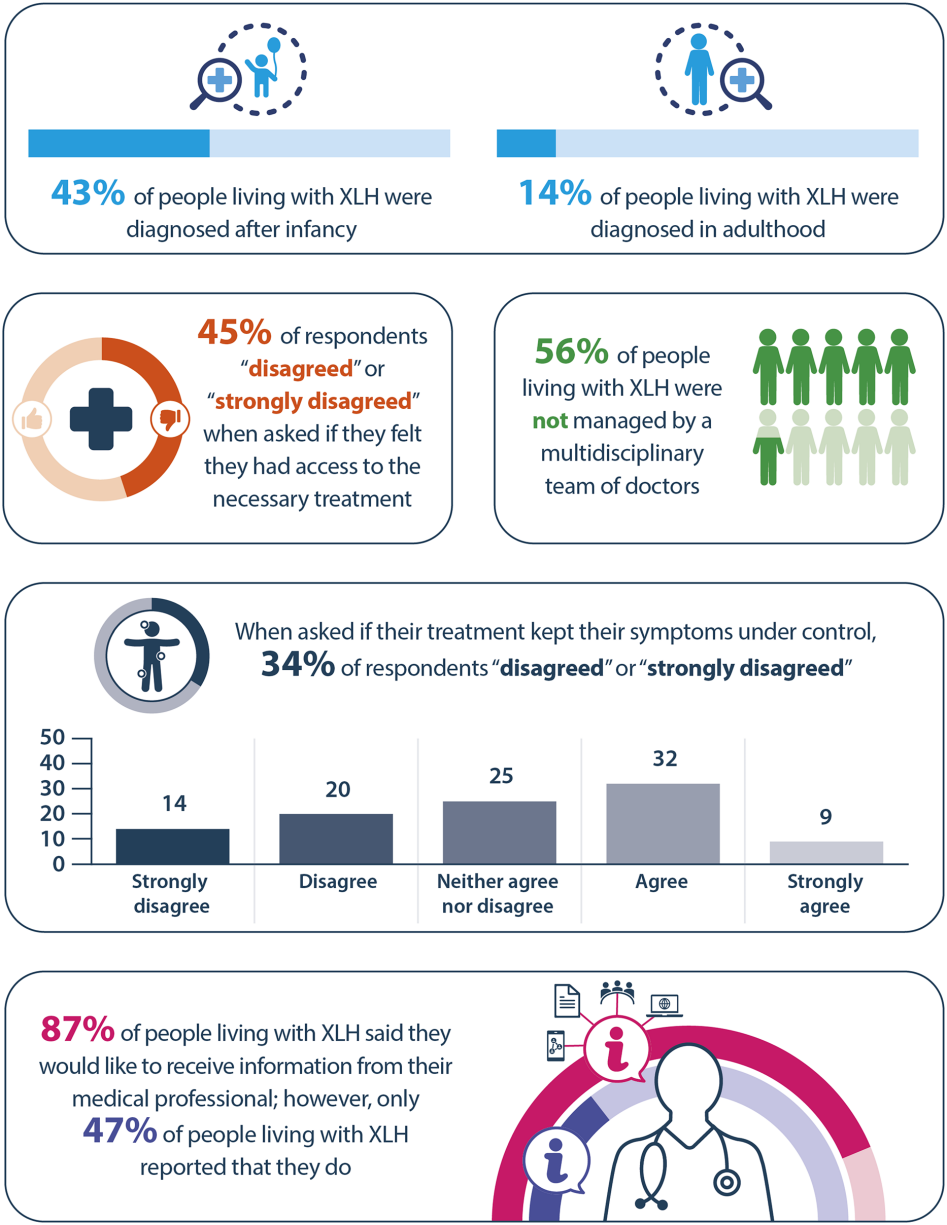
Summary of results from the IXLHA survey of 350 adults (≥18 years old) living with XLH from 35 countries. IXLHA, International XLH alliance; XLH, X-linked hypophosphataemia

The IXLHA developed an online survey to assess if current standards of care were aligned with Haffner et al.’s recommendations [7, 13]. This survey, conducted between 1 August 2021 and 11 September 2021, was completed by 350 adults (aged ≥ 18 years) with XLH from 35 countries, representing the largest known survey on XLH to date (Fig. 2) [13].

Haffner et al. recommended that patients be seen on a regular basis by an MDT involving clinicians, physiotherapists, dentists and social workers, organised by an expert in metabolic bone disorders, in addition to liaising with patient group representatives [7]. However, only 30% of respondents to the IXLHA survey were managed by a team of HCPs with different specialties (Fig. 2) [13]. It has been suggested that a lack of MDTs in rare bone disorders, such as XLH, stems from limited awareness of the importance of an MDT and limited numbers of rare bone disease experts in some regions [14]. In addition, many patients lose access to MDT care during their transition from paediatric to adult care services [14]. The results of this survey highlighted that the establishment of an MDT is a gap in the management of patients with XLH, which needs to be addressed, particularly during transition.

Patient opinions on the treatment of XLH were mixed. A third of patients (34%) reported that the treatment provided was not sufficient to keep symptoms under control and nearly half of patients (45%) felt they did not have access to the necessary treatment (Fig. 2) [13]. Attendees at XLH Matters 2023 suggested that this may be due to the absence of an MDT to manage the varied manifestations of the disease appropriately, a lack of HCP disease knowledge and the absence of country- or region-specific coordinated organisations and resources for rare bone diseases. Personalised treatment plans, delivered by an MDT, are ideally based on each patient’s specific clinical manifestations, needs and medical history [7]. In particular, for children with XLH about to undergo corrective surgery, treatment plans should be discussed in an MDT setting to ensure optimal pre-emptive medical treatment to prevent recurrence and facilitate rehabilitation post-surgery, including pain management, access to mobility devices and physiotherapy [7, 15]. It was agreed that people living with XLH need clear, specific guidance on the treatment and management of their symptoms, and how they receive this information is tantamount to building their confidence in navigating their treatment journey.

There is an evident disparity between the sources from which patients get their information vs. the desired source of information. According to the IXLHA survey, the majority of patients (87%) said they would like to receive information from their HCP; however, only 47% of respondents said they did (Fig. 2) [13]. This lack of curated information provided by HCPs is also prevalent in other rare bone disorders, such as osteogenesis imperfecta and fibrous dysplasia, and may be due to limited awareness, time restraints or the limited availability of materials among the HCPs themselves [14]. The causes may be medical schools providing insufficient educational materials on rare bone disorders to students, and organisational restrictions regarding patient-facing time, which may further contribute to the lack of disease awareness [14]. The discrepancy in how patients receive information vs. their desired source of information highlights the importance of raising awareness of XLH among HCPs and, subsequently, providing patients with more up-to-date and relevant information on the management of their condition.

### Supporting patients with XLH

A focus of discussion at XLH Matters 2023 was the changing burden of XLH at different life stages [7, 16]. During childhood, medical care is typically directed and/or guided by parents or guardians. However, adult patients need to actively manage care themselves, ask the right questions and seek appropriate clinicians to treat their condition. The attendees noted that young adults may lose access to MDT care. There are several factors that may lead to young adults being lost to follow-up during transition to adult care, such as the increased socioeconomic demands at this stage of life. This includes further education, family planning and relationships, which are in direct conflict with the time and resources needed to manage their condition. Young adults can often feel socially isolated from their peers who do not face the same challenges that a chronic disease brings [17]. This can also impact the aspirations of people living with XLH, such as making the decision to have children, which can carry a heavy hereditary burden as they may not want their child to face the same clinical manifestations throughout life [18]. Attendees reported that many of their patients felt unable to be the parent they aspire to be due to chronic pain, limited mobility and overall poor quality of health.

### Improving quality of appointments and information

**Fig. 3:**
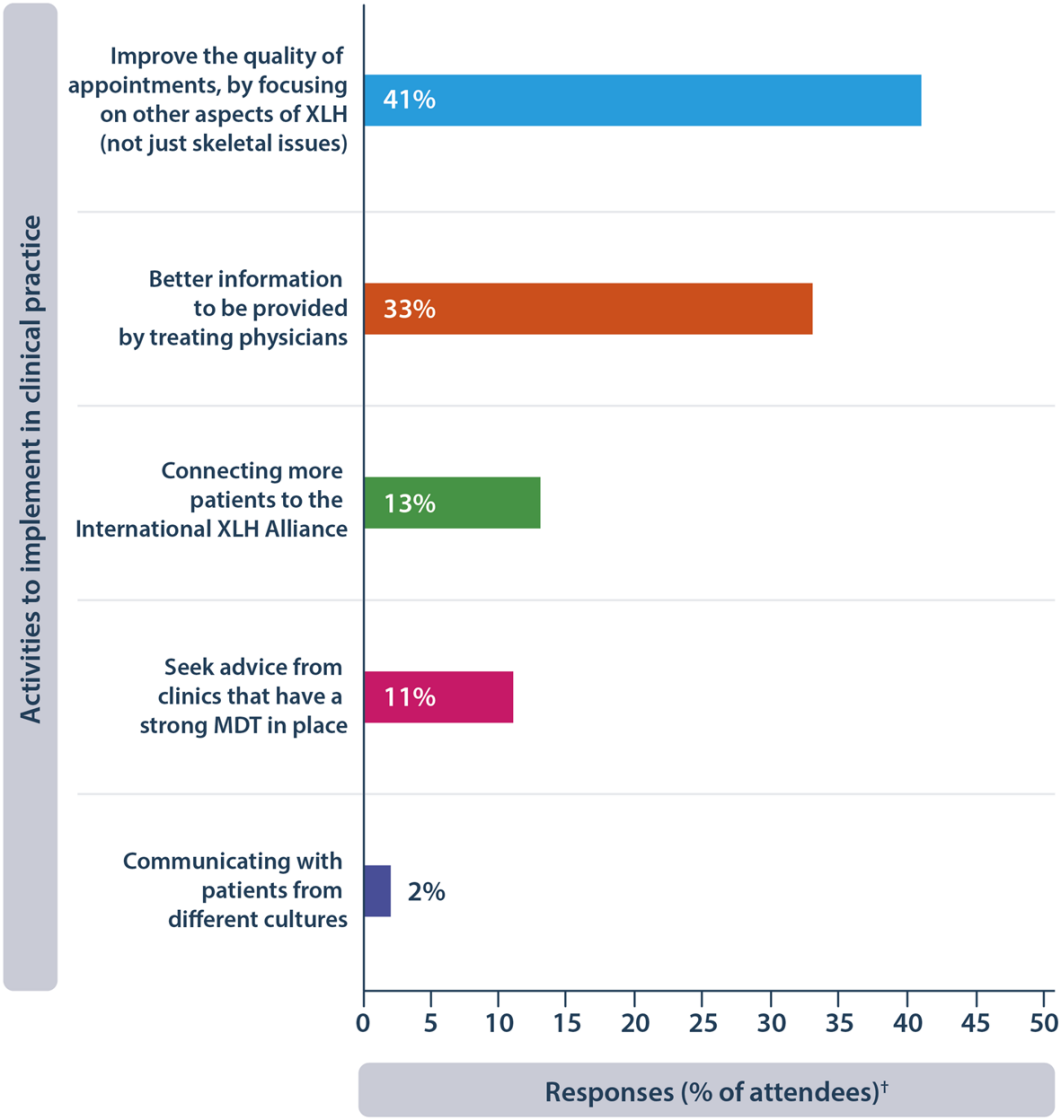
Attendees voted on which activity they thought was the highest priority to implement in clinical practice to support people with XLH and their families. ^†^Based on 102 responses. MDT, multidisciplinary team; XLH, X-linked hypophosphataemia

A range of activities were provided by Oliver Gardiner at XLH Matters 2023 that could help clinicians to support people with XLH and their families. Attendees at XLH Matters 2023 were asked to vote on the key activities that the clinical community could implement in practice (Fig. 3).

Improving the quality of appointments by broadening the discussion to focus on other aspects of XLH and not just skeletal manifestations was the top response. Extraskeletal manifestations of XLH are diverse and include hearing loss, enthesopathy and muscle weakness [7]. A UK study found that people with XLH have an increased prevalence of endocrine (OR = 3.46 [95% CI: 1.44, 8.31]) and neurological disorders (OR = 3.01 [95% CI: 1.41, 6.44]) than those in non-XLH cohorts [19]. Mental health conditions were also elevated in people with XLH, with depression rates being at least twice as high in people with XLH vs. those without (OR = 2.95 [95% CI: 1.47, 5.92]) [19]. These findings emphasise the widespread impact of XLH and the importance of having specific MDT members available to give people with XLH better information about managing their manifestations.

Regular MDT meetings are recommended to ascertain the best approach to manage the varying disease course and manifestations associated with XLH in a timely manner [7, 15]. Attendees highlighted that patients often only meet with one HCP from a single specialty once every 6 months, who may find it difficult to address all their manifestations and give the patient the information they need. In addition, patients may have limited access to the specialists required to manage the full clinical manifestations of the disease, including dental experts, ear, nose and throat (ENT) specialists, nephrologists, psychologists, and surgeons. However, it was agreed that it is not necessary for people with XLH to consistently see all MDT members throughout the entire disease course as manifestations can vary between patients and change in severity at different life stages. Select meetings with the appropriate specialists can ensure that all patient questions are answered and can help reduce the burden of frequent appointments for people with XLH. A recent study aimed to determine if a patient support programme (PSP) could help people with XLH cope with their condition [20]. A total of 59 participants were enrolled and most reported an improvement in HRQoL in all the examined dimensions, including, physical, emotional, social and school by Month 12 [20]. The study suggested that PSPs may improve HRQoL for people with XLH through education, therapy adherence, motivational interviews and frequent follow-up [20].

### Connecting patients to overcome cultural differences and challenges

Cultural differences were a key challenge discussed at XLH Matters 2023 as it was agreed that they can impact the quality of care received by patients with XLH and hinder the formation of an MDT. Language barriers were discussed as a factor that can cause difficulties for patients when communicating their symptoms to HCPs. Cultural differences were also flagged as a barrier to accessing care in rare bone disorders at the first Rare Bone Disease Summit, which took place virtually over 2 days in November 2021 [14]. For example, patient advocacy groups may not be available in some regions, which can limit access to high-quality, localised information [14]. In some cultures, stigmatisation resulting from the physical appearance of patients with rare bone disorders may prevent adults from seeking care for themselves or their children for fear of being ostracised [14]. It was agreed that these limitations lead to inconsistencies in care, which is damaging to patients and leaves them feeling uninformed and uncared for. In addition, in his talk at XLH Matters 2023, Oliver Gardiner, highlighted the unconscious bias in the workplace, as adults with XLH are overlooked for promotions due to a misperception of unreliability. Connecting patients to advocacy groups, such as the IXLHA, can help signpost people with XLH to support groups and to learn from each other’s experiences and therefore feel less isolated. Attendees supported the notion of creating pathways to improve patient outcomes, regardless of where they live, by utilising social media and developing telemedicine platforms, to share experiences and resources to unite the patient voice.

### Summary

The formation of an MDT to comprehensively address the varied clinical manifestations of XLH remains a challenge in the management of XLH and other rare bone disorders. As a result, patients can feel ill-informed and isolated, particularly during formative and transitional periods of their life, such as the adolescent years. It is clear from the results of the IXLHA survey that people living with XLH want: (1) more information from a range of specialists about their condition and; (2) regular meetings with an established MDT, with one core physician overseeing their treatment journey as a priority. Patient advocacy groups, such as the IXLHA, are a key resource to help address issues faced by people living with XLH and enable them to use their own voice to improve the care pathway.

## Optimising outcomes—improving health-related quality of life for people living with XLH

PROMs are valuable tools to assess symptoms, measure HRQoL and evaluate treatment success in terms of what is most important to patients and, importantly, is information received directly from the patient. There are several measures to assess various aspects of XLH (Table 2). PROMs have been used to measure the impact of burosumab treatment in people with XLH in clinical trials [6, 21, 22]. By implementing PROs in routine clinical practice, clinicians can monitor the symptoms, function and HRQoL of their patients, to ascertain whether patient outcomes are improving with their treatment regimen.

**Table 2:** Selected PROs and functional assessments used for monitoring people with XLH^†^

Specific outcome measure	Measures assessed	Age range	Time to complete
**HRQoL measures**			
EuroQol-5 Dimensions (EQ-5D) (3 versions: 3L, Y-3L, 5L) [23].	Five questions covering five domains: mobility; self-care; usual activities; pain/discomfort; and anxiety/depression with the Y-3L version specifically designed for self-completion by children and adolescents aged 8–15 years [23].	Children (8–15 years old) and adults [23].	2–5 min [24].
Short-Form 36/SF-36 v2 [25, 26].	Two summary scores of physical and mental wellbeing and 8 domain scores: Vitality, physical functioning, role physical, bodily pain, general health, role emotional, social functioning and mental health (36 questions) [25, 26].	Children (≥ 14 years old) and adults [27].	20–30 min [28].
**Measures to evaluate defined domains of health**			
Patient-Reported Outcomes Measurement Information System (PROMIS) [29].	Pain interference, fatigue and physical function mobility [29].	Children, (8–17 years old), parent-proxy (5–17 years old) and adults [30].	~3 min [31].
WOMAC [32].	Assesses pain, stiffness and physical function during various activities of daily living, 24 items [32].	Adults [25].	20 min [28].
**Pain measures**			
BPI [32].	Pain severity: Worst, least, average, current Interference: Mood, work, general activity, walking, relationships, enjoyment of life and sleep (15 items) [32].	Children and adults [33].	10 min [28].
**Functional assessments**			
6-min walk test (6MWT) [34].	Timed walk on 30 m track, standardised physical assessment [34].	Children and adults [35].	6 min [34].
Timed-Up-and-Go (TUG) [36].	Stand from chair, walk 3 m and back and return to sit down [36].	Children and adults [37].	N/A

^†^Use of these outcome measures in terms of preference will vary between clinicians, regional/country preferences and availability of tests

### The patient-reported experience of XLH

The rarity of XLH means there are few large-scale studies focusing on the patient-reported experience. Cheung et al. 2021 describes an analysis of data collected from a large multinational online survey to explore symptoms, complications and the overall experience of living with XLH as reported by the patients themselves [17]. The responses of 209 adults and 86 children/adolescents (proxy report) were analysed across eight age groups, which identified three main themes: [17].

1. Clinical signs and symptoms of XLH
2. Impacts of clinical signs and symptoms
3. Negative treatment experiences

Clinical signs and symptoms of XLH, including pain, stiffness and skeletal pathology, were present throughout life in people with XLH [17]. Pain was the most frequently reported manifestation of XLH experienced throughout the life course [17]. In children and adolescents, bone pain was frequently observed (33–40%); however, in adults, joint pain became more prominent than bone pain (34–45%) [17]. Severe pain was the most prominent symptom of XLH in adults, with pain reported upon waking up, during menstruation and after physical exercise [17]. Stiffness, however, became more common in adolescence than in childhood and increased during adulthood [17]. Dental problems were also present, including dental abscesses, tooth loss and pain [17]. Cheung et al.’s analysis showed that, among people living with XLH, the burden of pain changes throughout life and the location where the pain has the greatest impact alters with age [17]. Hence, PROMs are a useful tool to reveal patient concerns and manage them appropriately.

The impact of clinical signs and symptoms associated with XLH can have profound emotional and societal consequences [17]. Depression, anxiety and frustration were reported in adults with XLH as the burden of the clinical manifestations accumulates [17]. People with XLH in the UK were twice as likely to suffer from depression than their peers without XLH [19]. Similar results were also noted in Spain, where children with XLH reported moderate anxiety and depression with higher levels observed in adults [38]. This indicates that the burden of XLH is not only limited to the physical manifestations of the disease, but also the psychosocial issues that arise as a result. Involving a psychologist or patient support group in the treatment journey can help to address these issues.

Negative experiences with treatment and surgery were seen across all age groups but most notably in children and adolescents [17]. In children and adolescents, negative experiences were related to the frequency of oral phosphate whereas, for adults, the main burden was the side effects of treatment [17]. The burden of frequent phosphate dosing (4–6 times a day) has been suggested to likely contribute to poor adherence, which could lead to the progression of physical manifestations and further the disease burden [39]. Adults frequently reported difficulty accessing the appropriate treatment [17]. They noted frustrations with their doctors and dentists who seemed to have a lack of knowledge and understanding about XLH and available treatments [17]. To check if patients are adhering to treatment, it is important to ask relevant questions and use PROMs to indicate if treatment is working or not and to address the specific needs of each patient.

### The clinical perspective on PROMs

PROMs provide a valuable insight into the treatment journey from a patient’s perspective and highlight key areas where clinicians can improve their knowledge of the patient and personalise the care they are planning. Prior to attending XLH Matters 2023, HCPs were asked in a survey whether they agreed that it is important to measure HRQoL in people living with XLH. 95% of respondents “agreed” or “strongly agreed”; however, at the meeting, 86.6% of attendees admitted when polled that they do not routinely measure HRQoL in their clinical practice. To understand how HRQoL is measured in clinical practice, attendees participated in a workshop where they were tasked with answering which PROMs they currently used, which they thought should be measured and the barriers they faced in performing these measurements (Box 3).

**Box 3:** Understanding how patient experience is measured in clinical practice

**Fig. 4:**
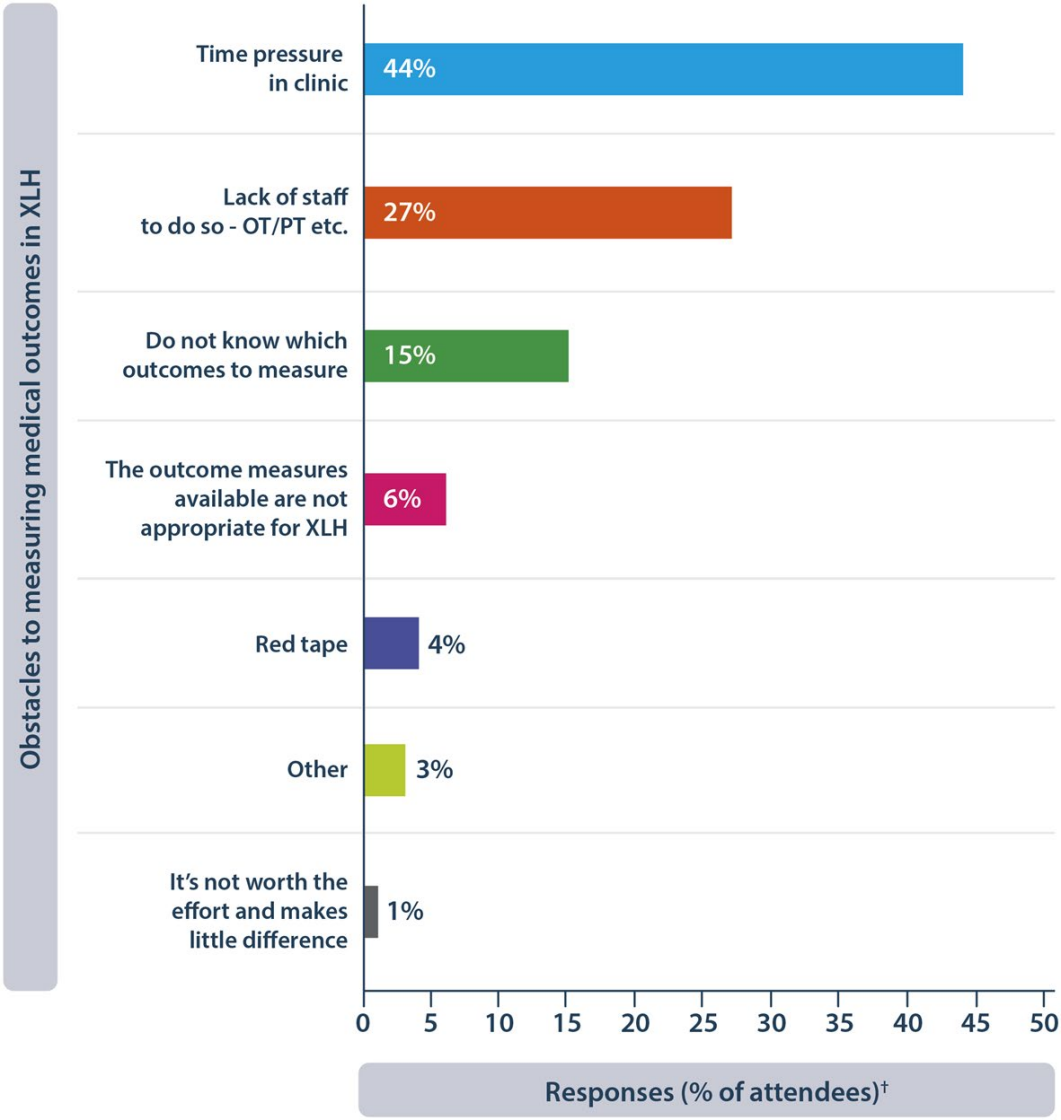
Attendees were asked to vote for their top 2 obstacles to measuring medical outcomes of XLH in clinical practice. ^†^Based on 203 responses. OT, occupational therapist; PT, physical therapist; XLH, X-linked hypophosphataemia

**Current measures of symptoms, mobility and HRQoL in clinical practice**
For pain, the most frequently reported measure used by attendees were the BPI, Visual Analog Scale (VAS) and FACES pain scale; however, literature suggests that the FACES pain scale may not reliably assess the burden of pain in children with XLH [29].
Some attendees reported using no specific scale, asking the patient arbitrary questions or relying on the patient to keep a pain diary
For mobility, the 6MWT was the most reported outcome measured by HCPs in people with XLH
The Pediatric Quality of Life Inventory (PedsQL) was also frequently used to measure the impact of XLH on daily life in children with XLH
**Preferred measures of symptoms, mobility and HRQoL in clinical practice**
Many attendees preferred using the BPI, PROMIS and EuroQol five-dimension questionnaire (EQ-5D) to measure pain
VAS was suggested by one respondent due to its ease of use, in addition to using WOMAC, which has been previously used in clinical trials of burosumab [6, 21, 22].
For mobility, the 6MWT and the Chair Rise test were suggested for use in routine clinical practice
The majority of respondents chose the EQ-5D or a variant of the Short-Form survey to measure emotional wellbeing
Other suggestions for measuring HRQoL included evaluating social interactions, sexual activity, education, dental health and financial burden on the family
**Barriers to measuring patient experience and proposed solutions**
44.3% of attendees at XLH Matters reported time as a barrier to measuring HRQoL (Fig. 4), followed by lack of staff to perform measurements (27.1% of attendees)
More personnel in the management team from a range of specialties, including physical therapists and psychologists, was proposed to overcome the limited time that the primary care provider has with each patient
Recruiting nurse coordinators was recommended by the expert faculty at the meeting to assist with these measurements

It is evident that there is a lack of consensus on which measures to use. Despite many attendees advocating the use of BPI and VAS, there were relatively few attendees who regularly used these in clinical practice, with some attendees relying on qualitative reports of pain. For measuring mobility, there was a higher level of agreement with most attendees using the 6MWT, although the Chair Rise test was also used in routine practice. The Timed Up and Go test was used in clinical practice by attendees to measure balance and risk of falling. There was substantial agreement on the use of the EQ-5D and the SF-36 survey for emotional wellbeing. Clearer guidelines would be beneficial to support the choice of PROMs and standardise their implementation.

Time and lack of personnel were the main barriers to measuring any PRO including HRQoL in clinical practice (Fig. 4). One delegate reported that some of the mobility tests are administered by physical therapists and, therefore, may not be available in all clinics. This supports the importance of establishing an MDT for XLH. Another delegate answered that “quick and easy is the priority” when choosing a method for measuring HRQoL. Time has previously been cited as the main barrier to performing PROMs with suggestions that it may be due to the survey length and that shorter surveys would be preferable [40, 41].

Reflecting on these barriers, attendees were asked to list the changes they would implement into clinical practice as a result of this workshop (Box 4).

**Box 4:** Summary of changes attendees would make to their clinical practice

Use a questionnaire and/or a more structured system
Improve current questionnaires, for example, by standardising or digitalising to facilitate completion
Incorporate routine measurements of PROMs as part of regular clinic appointments
Implement a checklist to ensure that all appropriate outcomes are measured
Improve HCP knowledge and educate patients on the importance of outcome measures
Work with and learn from interdisciplinary colleagues (e.g. psychologists and physiotherapists) to measure functional outcomes

Following XLH Matters 2023, according to the post-meeting survey, there was a 30% increase in respondents agreeing and strongly agreeing to feeling confident in measuring HRQoL for people living with XLH. More awareness of the importance of PROMs has resulted in increased confidence, which may be reflected in the use of PROMs in clinical practice. To further facilitate the use of PROMs, implementation of PROMs in the medical record software by hospitals could be beneficial.

### Summary

The burden of XLH varies throughout life [17]; hence, it is important to measure the experience of people living with XLH on a regular basis. This ensures that, at each stage, the most prominent concerns of the patient are identified and addressed, with the ultimate goal of improving their HRQoL. PROMs are the best approach to provide information on the emotional and social burden of XLH and help guide clinical decision-making. Routinely applying PROMs remains a challenge in XLH, despite most attendees recognising their importance for prompt and effective disease management. Limited time and personnel are the top two obstacles to implementing PROMs in clinical practice. However, the establishment of an MDT, involving physical therapists, psychologists and nurse coordinators, in addition to the development of more structured, standardised and digital questionnaires that are easy to complete, may help with completion rates. Raising awareness of the importance of PROMs among HCPs and patients is instrumental to ensure that they are effectively implemented to improve quality of care for all people living with XLH.

## Collaborating as a multidisciplinary team

**Fig. 5:**
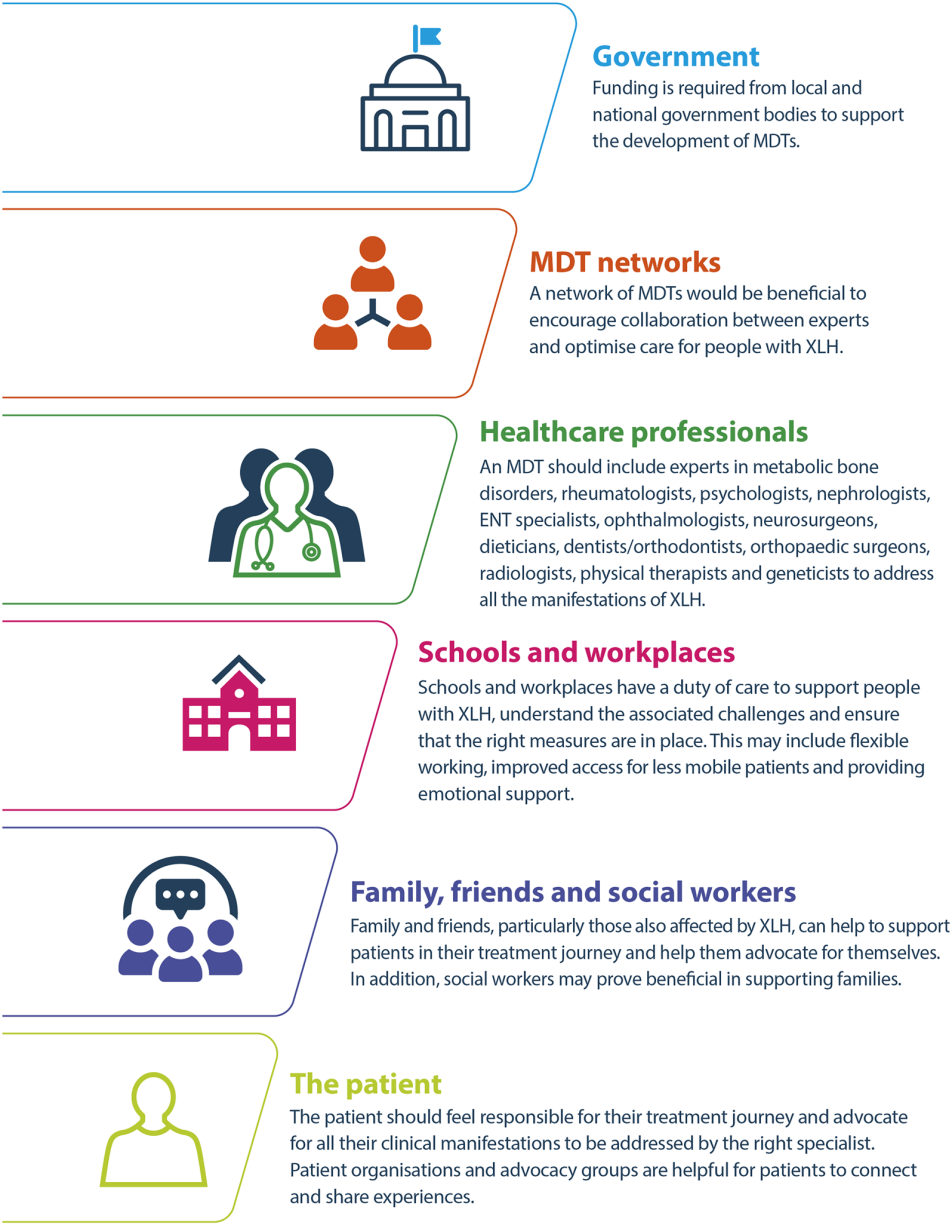
A representation of the levels of support needed for the successful management of the diverse clinical manifestations of XLH, listed in order of size from largest (top) to smallest (bottom). ENT, ear, nose and throat; MDT, multidisciplinary team; XLH, X-linked hypophosphataemia

People living with XLH experience a diverse range of clinical manifestations, with chronic hypophosphataemia having a detrimental effect on bone, teeth, muscle, joints and hearing [7, 42]. Manifestations usually begin to develop in the first or second year of life and continue to accumulate throughout the lifetime in both children and adults with XLH, leading to a profound impact on their HRQoL [7, 25, 42, 43]. To ensure that people living with XLH receive the best lifelong care, individually optimised management strategies, involving a network of multidisciplinary specialists to manage these manifestations, is essential, including specialists to address manifestations, such as nephrocalcinosis and hyperparathryoidism, that may arise during the course of treatment. Management of XLH should be supported at every level of the patient’s life from friends and family, to schools and workplaces with financial support from local and national government bodies (Fig. 5).

**Fig. 6:**
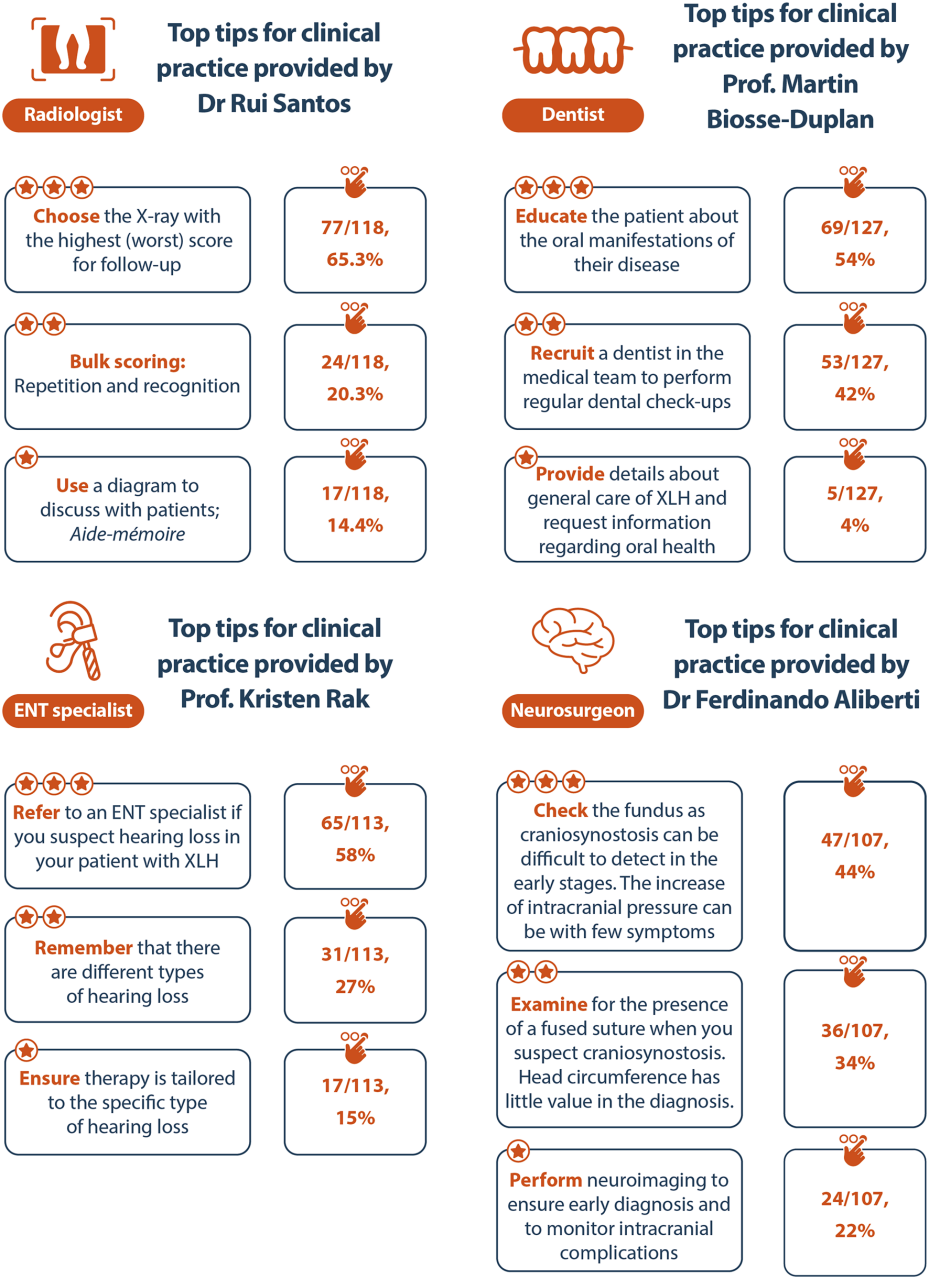
Top tips for clinical practice in each speciality as voted for the attendees at XLH Matters 2023. ENT, ear, nose and throat; XLH, X-linked hypophosphataemia

At XLH Matters 2023, four MDT experts, a dentist, radiologist, ENT specialist, and neurosurgeon, shared their expertise and experience of working in an MDT. This section summarises key takeaways from each of the ‘Meet the XLH expert’ workshops and explores strategies to encourage effective collaboration within an MDT. A summary of the top tips provided by MDT experts is shown in Fig. 6.

### Radiologist

Chronic hypophosphataemia can lead to the development of rickets in children, resulting in the characteristic bowing of lower limbs and waddling gait, growth plate abnormalities and short stature [25]. In adults, unresolved hypophosphataemia can lead to the progression of osteomalacia, increasing the risk of pseudofractures, osteoarthritis and enthesopathy [25]. A global online survey of 322 adults with XLH or caregivers to children with XLH revealed the lifelong burden experienced by people living with XLH and associated skeletal abnormalities [25]. For example, lower limb abnormalities, specifically bowing of the tibia and/or fibula were reported in 72% of children (65/90) and 77% of adults (178/232), whereas short stature was found in 80% (72/90) and 86% (200/232) of children and adults respectively [25]. A history of fractures was also prevalent in adults living with XLH at 44% (102/232) [25].

In addition to bowing of long bones, radiographic features of rickets include fraying (indistinct margins of the metaphysis), splaying (widening of metaphyseal ends) and cupping (concavity of metaphysis) of bones [44]. When assessing rickets, the use of RSS grade definitions as described by Thacher et al. is recommended [45]. Notably, an RSS tool of the wrist and knee is currently in development and will provide HCPs with an additional resource to aid rickets severity scoring in children with XLH.

The top tips identified by the radiologist Dr Rui Santos (Evelina London Children's Hospital and Guy's and St Thomas’ NHS Foundation Trust, London, UK) at XLH Matters 2023 are summarised in Fig. 6. These include using the X-ray with the highest score (i.e. worst) for follow-up and utilising an *aide-mémoire* to facilitate communication with patients. The benefits of blind and bulk scoring to reduce potential treatment bias was also highlighted. Given the plethora of radiological manifestations associated with XLH, a radiologist represents a key member of the MDT. Thus, HCPs are encouraged to regularly communicate with a radiologist and find an approach that works for their centre.

### Dentist

Dental abnormalities are also prevalent in people living with XLH [43]. In Skrinar et al.’s global survey, dental abscesses were reported in 51% (46/90) of children and 82% (189/232) of adults, highlighting the progressive impact of XLH on oral health [25]. The accumulation of these oral manifestations places a substantial burden on people living with XLH. Children and adults with XLH often experience pain, physical discomfort, premature tooth loss and functional limitations, severely impacting their oral HRQoL (OHRQoL). Of note, median scores evaluating OHRQoL were significantly worse in an XLH cohort compared with those living with another rare skeletal disease, osteogenesis imperfecta (OI) [46]. For example, median functional limitation scores were higher (worse) in adults with XLH: 6.5 compared with 4.0 in the OI cohort (*p* < 0.05) [46]. A qualitative, rather than quantitative, evaluation of OHRQoL in children and adults with XLH revealed a frequently chaotic oral healthcare pathway with consequences for their social, professional and school integration [47].

Treatment with oral phosphate and active vitamin D has been shown to reduce endodontic infections and the severity/frequency of periodontitis in children and adults with XLH [48–50]. Clinical data regarding the impact of burosumab treatment on the oral manifestations of XLH remains limited, however, improvements in oral health have been reported in a study of burosumab [51]. In a recent study, the mean number of dental abscesses were significantly reduced in children treated with burosumab compared with those receiving oral phosphate and vitamin D (*p* = 0.04) [51]. In addition, in a post-hoc analysis of a 64-week, open-label, randomised controlled study in children with XLH receiving burosumab treatment, dental abscesses were not reported in younger children (< 5 years old) but were reported in 53% of older children (5–12 years old) [52].

Given the extensive dental abnormalities experienced by people living with XLH, efforts are needed to optimise the oral care pathways and outcomes. The burden of poor oral health faced by patients with XLH was emphasised by attendees, highlighting the need to raise the awareness of the oral manifestations associated with XLH, as well as ensuring regular check-ups by a dentist within an MDT. Indeed, these were among the top tips provided by the dentist Prof. Martin Biosse-Duplan [Bretonneau Hospital (AP-HP) and Paris Cité University, Paris, France] at XLH Matters 2023 (Fig. 6).

### ENT specialist

Hearing loss may also affect people living with XLH and has been observed in children as early as 11 years of age [7, 43]. The severity and prevalence of hearing loss varies depending on age, however, sensorineural (caused by problems in the cochlea) and conductive (caused by problems with the middle ear) or mixed (affecting inner and middle ear) hearing loss has been reported in up to 76% of adults with XLH [43].

Diagnostic tools, such as the Weber and Rinne tests and tympanometry, are useful to assess hearing loss. Weber and Rinne tests are performed using a tuning fork and determine if hearing loss is associated with sensorineural or conductive hearing pathways. A tympanogram provides a graphical reading of the eardrum functioning in response to alterations in air pressure within the ear canal and the evaluation of otoacoustic emissions.

Treatment for the different forms of hearing loss include middle ear surgery and hearing aids or cochlear implants; offering these treatments to people with XLH experiencing hearing loss may greatly improve their HRQoL. Thus, if any level of hearing loss is suspected, the referral of patients to an ENT specialist is recommended (Fig. 6). Top tips provided by the ENT specialist Prof. Kristen Rak (University Hospital Würzburg, Würzburg, Germany) at XLH Matters 2023 emphasised the importance of raising awareness of the different forms of hearing loss associated with XLH and the tailored treatments that are available.

### Neurosurgeon

Children with XLH may exhibit cranial abnormalities; for example, craniosynostosis has been reported in ~60% of children, with most cases associated with the premature fusion of sagittal sutures [43, 53, 54]. Despite a higher prevalence of XLH in women, a higher incidence and severity of craniosynostosis is observed in men, potentially highlighting a protective effect of heterozygous phosphate-regulating endopeptidase homologue on the X chromosome (*PHEX)* mutations that are present in women [54].

While common features of craniosynostosis include premature fusion of the sagittal sutures, scaphocephaly and altered head size/circumference, diagnosis in children with XLH can be difficult [54]. Routine screening to detect any potential cranial shape anomalies is recommended in the first five years of life [54]. Once features of craniosynostosis have been recognised, prompt neurological, ophthalmological and neuroradiological assessments are necessary. Ultrasound, 3D computed tomography (CT), magnetic resonance imaging (MRI) and fundoscopic examination have been identified as useful diagnostic tools. If left untreated, craniosynostosis can cause permanent skull deformities, increasing the risk of further complications, such as elevated intracranial pressure (ICP), Arnold Chiari Malformation (ACM) and papilloedema [54]. ICP may be associated with few/mild symptoms, routine fundoscopic assessment was therefore recommended among the top tips from the neurosurgeon Dr Ferdinando Aliberti (Santobono-Pausilipon Children’s Hospital, Naples, Italy) at XLH Matters 2023 (Fig. 6).

Treatment with oral phosphate and active vitamin D does not appear to prevent the development of craniosynostosis in infants with XLH [54]. The natural evolution of the neurological complications (e.g. syringomyelia) is still unknown. Thus, current treatment options usually involve cranial surgery to relieve pressure on the brain. Given the importance of prompt treatment, effective collaboration between paediatricians and neurosurgeons was specifically emphasised during discussions at XLH Matters 2023.

### Practical tips and collaborating with an MDT

In addition to sharing their clinical experience, the MDT experts individually provided their three top tips (Fig. 6). Attendees at XLH Matters 2023 were asked to vote for the top tip that they would apply in their clinical practice, with responses recorded on workmats. The top tips for each MDT as voted by the attendees are summarised in Fig. 6.

Of the top tips provided by the radiologist at XLH Matters 2023, 65% of attendees voted ‘Choose the X-ray with the highest (i.e. worst) score for follow-up’ as the tip that they would apply in their clinical practice (Fig. 6). Attendees noted the importance of practical tools to aid rickets severity scoring in patients with XLH, with the development of a new diagrammatic aid that will be available in the future.

Given the diverse range of clinical manifestations that people with XLH experience, it is not surprising that ‘Educate the patient about the oral manifestations of their disease’ (54%, 69/127) was voted as the top dentist tip. Similarly, referring patients to an ENT specialist immediately if hearing loss is suspected and the use of fundoscopic examinations in monitoring craniosynostosis were also voted as top tips by attendees. Highlighting potential knowledge gaps among HCPs and the importance of using specific MDT specialists can provide patients with valuable insights regarding the management of their manifestations.

HCPs were asked how feasible it was for them to collaborate with a dentist, radiologist, ENT specialist, and neurosurgeon (1 = not feasible, 5 = very feasible) before and after attending XLH Matters 2023. Importantly, there was an increase in the number of HCPs who felt that they were able to better collaborate with all members of the specified MDT specialists following XLH Matters 2023. Specifically, there was a 64% increase in respondents who felt that they were able to collaborate better with a dentist, followed by a 43% increase for collaborating with an ENT specialist. There was a 23% and 8% increase in HCPs who reported that they felt better able to collaborate with a neurosurgeon and dentist, respectively. A high number of HCPs felt it was ‘very feasible’ to work with a radiologist prior to the meeting (53%, 65/122). While barriers to the coordination of an MDT may exist in certain centres, continued efforts to increase the collaboration between MDT specialists are necessary (Box 5). Increasing the awareness of XLH among HCPs, as well as defining the XLH MDT structure, were identified as key strategies to improve the coordination of MDT networks and optimise outcomes for people living with XLH.

**Box 5:** Defining an MDT

A discussion at XLH Matters 2023 highlighted variations in terminology across different countries to describe groups of specialities managing the care of patients with XLH, which may limit collaboration
Optimal treatment strategies for patients with XLH require the coordination of a multitude of specialists throughout their lifetime (Fig. 5). As noted by attendees, certain specialists including nurses, physiotherapists and psychologists may not be considered part of the immediate XLH MDT in some regions. Concerted efforts to define the MDT network involved in the management of XLH is therefore encouraged, to ensure patients living with the disease can access the appropriate lifelong specialist care that they require.

### Beyond skeletal manifestations of XLH

A focus of discussion at XLH Matters 2023 was the need to educate patients on the diverse range of clinical manifestations that they may experience beyond skeletal manifestations. Oliver Gardiner, the chair of the International XLH Alliance (IXLHA), proposed a range of activities to help clinicians support people living with XLH (see section "Living with XLH—Our patients' perspectives"). Improving the quality of appointments with patients by focusing on other aspects of XLH, such as extraskeletal manifestations, was considered the top activity by attendees at XLH Matters 2023. Extraskeletal manifestations experienced by people living with XLH include hearing loss, enthesopathy and neurological disorders, such as ACM [7]. Access to specific MDT experts to identify and manage these manifestations throughout the lifetime of patients with XLH is therefore crucial to ensure positive outcomes. The paramount importance of increasing the awareness of the extraskeletal manifestations of XLH among HCPs and collaborating as an MDT across different centres was emphasised in a complex clinical case presented at this year’s meeting. In this case, opportunities to identify and address extraskeletal manifestations, namely ACM, were missed leading to the death of a 28-year-old male with XLH.

Systematic neurological assessments are necessary to ensure the prompt diagnosis of potential complications such as ACM in people living with XLH. Attendees supported this consensus, noting the importance of including ACM and other extraskeletal manifestations in training curriculums, as well as the development of standardised assessment tools. To avoid poor and preventable outcomes in patients living with XLH, concerted efforts are needed to raise the awareness among HCPS of the extraskeletal manifestations.

### Summary

Given the phenotypically diverse range of clinical manifestations that continue to accumulate throughout the lifetime of people living with XLH, clinical management strategies that involve a network of multidisciplinary specialists are necessary. This ensures that manifestations are promptly identified and addressed as the disease progresses, ultimately reducing the disease burden experienced by people living with XLH and improving their HRQoL. MDTs need to belong to a wider national network and connect with other MDTs with the support of local governing bodies to advocate for funding and improve standards of care. A knowledge gap still exists regarding the various extraskeletal manifestations of XLH, thus there is a need to raise awareness of the less commonly known manifestations, to reduce the burden of disease, morbidity and mortality in people living with XLH.

## Supporting adolescents living with XLH

Adolescence is a universally challenging period of change including rapid psychosocial, cognitive and physical development. This life stage can be an even greater challenge for adolescents with XLH who face the same societal pressures as their peers, while also navigating the transition from paediatric to adult care and managing profound physical impairment.

Children and adolescents with XLH have reported skeletal pathology problems more frequently than adults with XLH, in particular, short stature and bone and joint impairment [17]. As a result, issues with physical exertion, such as difficulty running and frequent tiredness, have been reported more frequently than issues with movement [17]. The inability to participate in the same sports and activities as their peers because of XLH-related manifestations, can lead to adolescents experiencing concerns with their body image and emotional distress [16, 17]. This can single them out from their peers at a time in life when bodily appearance is important for self-esteem [17]. In ‘XLH Matters 2022: Insights and recommendations to improve outcomes for people living with X-linked hypophosphataemia’, the authors emphasised that people with XLH become more aware of their appearance throughout adolescence [1]. Adolescents with XLH have reported being bullied as a result of their physical appearance or inability to participate in the same activities as their peers [17]. Hence, it is important to address the clinical and emotional manifestations of XLH in adolescents to build their confidence and help them come to terms with their condition before moving into adult care [55].

A primary objective for effective transition from paediatric to adult healthcare services is to prevent loss to follow-up that may potentially result in poorer outcomes in adulthood; this can occur in many disorders, including XLH [56]. Factors that can contribute to loss to follow-up may include: a fear of a new hospital, inadequate preparation throughout childhood/adolescence, and a lack of engagement from adolescents [56]. Additionally, adolescents can find it difficult to leave behind the paediatric healthcare provider with whom they have established a relationship and trust [56]. HCPs themselves may also face challenges in ensuring a smooth transition, such as maintaining communication between paediatric and adult healthcare providers, training limitations and different practices between clinics [56].

There are currently only a few recommendations for effective transition of care to support HCPs, and there is no specific framework on how to implement a successful transition programme for XLH [1, 7, 55, 57]. Adolescents themselves should be encouraged to participate in the clinical decision-making process, rather than decisions coming solely from parents or HCPs, as they are now young adults and will shoulder the responsibility to continue the journey, sometimes on their own, into adulthood. Expert recommendations regarding transition from paediatric to adult care for adolescents with XLH include preparing young people for transition early, assessing transition readiness, encouraging collaboration between paediatric and adult clinicians, and introducing adolescents to their new team prior to transfer [1]. Dahir et al. published a timeline covering key aspects that should be considered before transition and 3–6 months after transfer to adult healthcare services [55]. More guidelines and resources with an improved awareness of the importance of transition may help standardise the transition process for all adolescents with XLH and prevent a break in long-term follow-up [58].

A survey of HCPs prior to attending XLH Matters 2023 indicated that transition was the most commonly identified gap in the management of people living with XLH. To further examine the importance of transition in XLH at the meeting, attendees were introduced to a case study prior to completing a workshop on how to improve the transition period from paediatric to adult care.

### Navigating the transition from paediatric to adult care

**Fig. 7:**
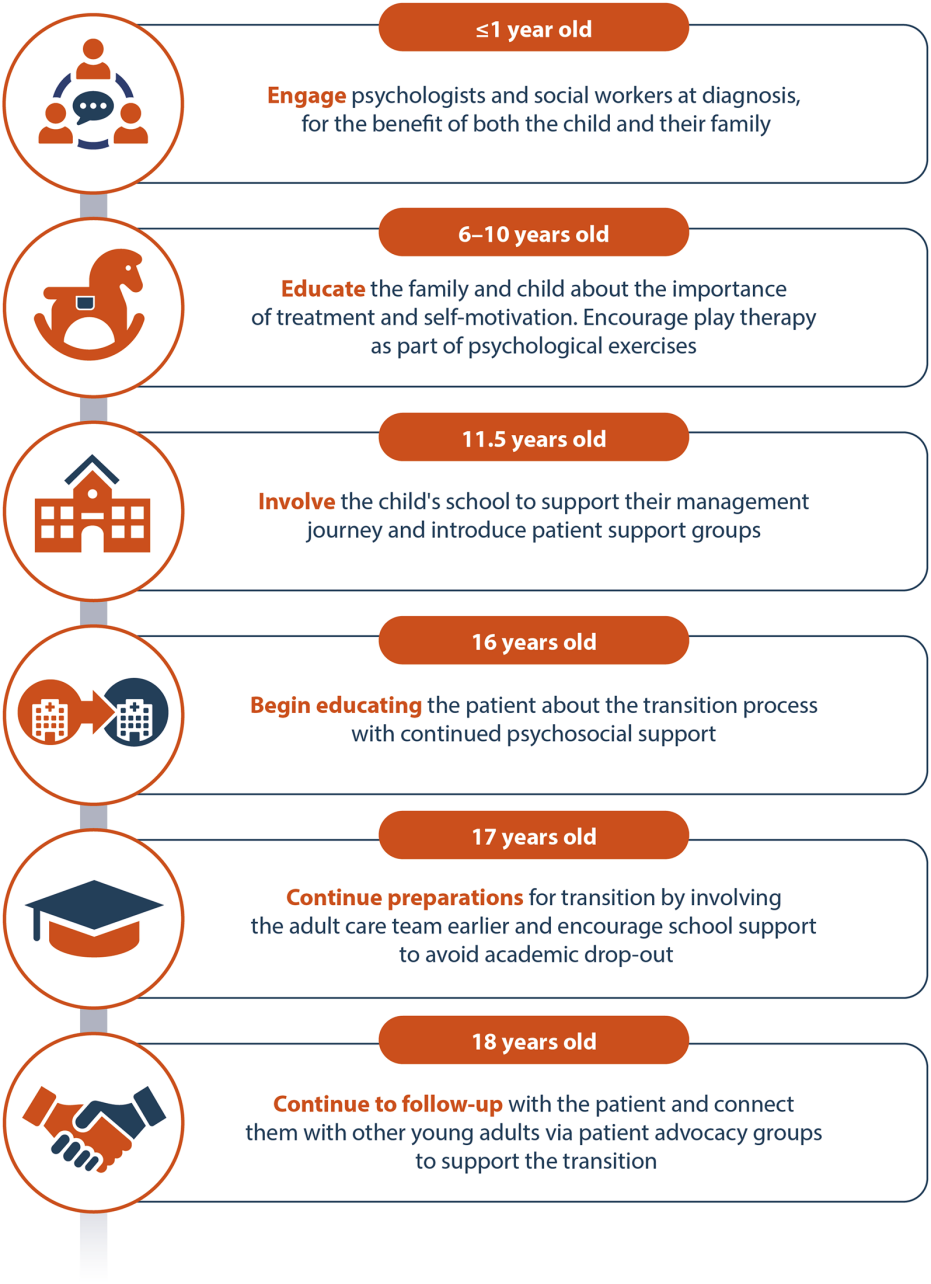
Proposed patient journey suggested by attendees at XLH Matters 2023 to support children and adolescents with XLH in their transition to adult care. XLH, X-linked hypophosphataemia

A case study of an adolescent living with XLH who was lost to follow-up during transition to adult care was presented. Following the presentation, attendees developed a timeline of the patient’s journey in a workshop session. The attendees were asked to consider new interventions that could have been initiated at each stage throughout childhood and adolescence to prevent the loss to follow-up during transition from paediatric to adult care (Fig. 7).

One of the key areas identified for intervention was the psychosocial burden of XLH which is prominent in adolescents [16]. Many attendees commented that engaging psychologists and social workers throughout childhood could benefit both the child with XLH and their family. In studies of chronic diseases, a patient- and family-centred approach was paramount for sustaining the effects of psychosocial treatment [59]. This could include play therapy to avoid treatment-related anxiety experienced by the child, in addition to connecting children with XLH and other rare chronic diseases with their peers via patient advocacy groups. The integration of telemedicine can help connect patients to each other and to HCPs they might not otherwise be able to communicate with due to geographical barriers [55]. In their publication, Dahir et al. suggested connecting patients to advocacy groups at the age of 17 years as part of the transition plan [55]. This could be via the XLH Network or social media, such as Meta (formerly Facebook) [55]. Ensuring children with XLH are able to connect with and learn from their peers who experience the same burden could help them feel less isolated and more well informed.

Both patient and parental education has been recommended to encourage self-advocacy, and to guide parents on empowering their children to take ownership of their disease [55]. Encouraging adolescents with XLH to take ownership of their disease is crucial to ensure a smooth transition. Parents and guardians are no longer responsible for driving their care; ensuring that adolescents feel comfortable discussing their condition and management options is important to keep them engaged with their treatment journey. Dahir et al. encourages self-advocacy from as early as 12 years old to aid transition readiness [55], and there are also tools available to support patients and HCPs with transition. The XLH Network has developed a toolkit that can support children and adolescents with XLH to learn self-advocacy and manage their health while taking care of their social and emotional needs [60]. For HCPs, the Ready Steady Go programme provides three surveys that can be completed with children aged over 11 years, at different stages of the transition period, to help them take ownership of their needs and be fully informed before their transition [61]. Raising awareness of these valuable resources among HCPs will help facilitate those embarking on the transition to the adult care service.

### Summary

Transition to the adult care service can be a daunting prospect for adolescents with XLH. Their already high levels of psychological distress could be exacerbated by the unfamiliarity of this transition process. Effective transition from paediatric to adult care pathways is vital to ensure consistent and optimal management of XLH throughout life [58]. This highlights the need for evidence-based guidelines with a clear framework to support HCPs and their patients during the transition period and prevent loss to follow-up. This could include ensuring psychosocial support is available to the patients and their family at an early age and preparing them for the transition as soon as possible. A smooth transition is important to optimise clinical outcomes, maintain lifelong standards of care and improve quality of life.

## Data Availability

No datasets used in this supplement.

## Abbreviations

6MWT: 6-Min walk test
ACM: Arnold Chiari malformation
APHP: Assistance publique–hôpitaux de Paris
BFI: Brief Fatigue Inventory
BPI: Brief Pain Inventory
CT: Computed tomography
ENDO-ERN: European Reference Network on rare endocrine conditions
ENT: Ear, nose and throat
EQ-5D(-3L/-Y-3L/-5L): EuroQol-5 Dimensions (-3 level/-child-friendly-3 level/-5 level)
EU: European Union
FGF23: Fibroblast growth factor 23
HCP: Healthcare professional
HRQoL: Health-related quality of life
ICP: Intracranial pressure
IRCCS: Istituto di ricovero e cura a carattere scientifico
IXLHA: International XLH Alliance
LSDB: Locus-specific database
MDT: Multidisciplinary team
MRI: Magnetic resonance imaging
N/A: Not applicable
NHS: National Health Service
OHRQoL: Oral health-related quality of life
OI: Osteogenesis imperfecta
OLE: Open-label extension
OT: Occupational therapist
PedsQL: Pediatric quality of life inventory
PHEX: Phosphate-regulating endopeptidase homologue on the X chromosome
PRO: Patient-reported outcome
PROM: Patient-reported outcome measure
PROMIS: Patient-reported outcomes measurement information system
PSP: Patient support programme
PT: Physical therapist
RSS: Rickets severity score
SF-36: Short-form 36
TUG: Timed-up-and-go
VAS: Visual analog scale
WOMAC: Western Ontario and McMaster Universities Osteoarthritis Index
XLH: X-linked hypophosphataemia
